# A High-Throughput Phenotypic Screen of the ‘Pandemic Response Box’ Identifies a Quinoline Derivative with Significant Anthelmintic Activity

**DOI:** 10.3390/ph15020257

**Published:** 2022-02-21

**Authors:** Harrison T. Shanley, Aya C. Taki, Joseph J. Byrne, Abdul Jabbar, Tim N. C. Wells, Kirandeep Samby, Peter R. Boag, Nghi Nguyen, Brad E. Sleebs, Robin B. Gasser

**Affiliations:** 1 Department of Veterinary Biosciences, Faculty of Veterinary and Agricultural Sciences, The University of Melbourne, Parkville, VIC 3010, Australia; hshanley@student.unimelb.edu.au (H.T.S.); aya.taki@unimelb.edu.au (A.C.T.); byrnej1@unimelb.edu.au (J.J.B.); jabbara@unimelb.edu.au (A.J.); 2 Medicines for Malaria Venture (MMV), 1215 Geneva, Switzerland; wellst@mmv.org (T.N.C.W.); sambyk-consultants@mmv.org (K.S.); 3 Monash Biomedicine Discovery Institute, Department of Biochemistry and Molecular Biology, Monash University, Clayton, VIC 3800, Australia; peter.boag@monash.edu; 4 Chemical Biology Division, The Walter and Eliza Hall Institute of Medical Research, Parkville, VIC 3052, Australia; nguyen.n@wehi.edu.au (N.N.); sleebs@wehi.edu.au (B.E.S.)

**Keywords:** *Haemonchus contortus*, parasitic nematode, *Caenorhabditis elegans*, anthelmintics, small molecules, *Pandemic Response Box*, phenotypic screening

## Abstract

Parasitic nematodes cause diseases in livestock animals and major economic losses to the agricultural industry worldwide. Nematodes of the order Strongylida, including *Haemonchus contortus*, are particularly important. The excessive use of anthelmintic compounds to treat infections and disease has led to widespread resistance to these compounds in nematodes, such that there is a need for new anthelmintics with distinctive mechanisms of action. With a focus on discovering new anthelmintic entities, we screened 400 chemically diverse compounds within the ‘*Pandemic Response Box*’ (from Medicines for Malaria Venture, MMV) for activity against *H. contortus* and its free-living relative, *Caenorhabditis elegans*—a model organism. Using established phenotypic assays, test compounds were evaluated in vitro for their ability to inhibit the motility and/or development of *H. contortus* and *C. elegans*. Dose-response evaluations identified a compound, MMV1581032, that significantly the motility of *H. contortus* larvae (IC_50_ = 3.4 ± 1.1 μM) and young adults of *C. elegans* (IC_50_ = 7.1 ± 4.6 μM), and the development of *H. contortus* larvae (IC_50_ = 2.2 ± 0.7 μM). The favourable characteristics of MMV1581032, such as suitable physicochemical properties and an efficient, cost-effective pathway to analogue synthesis, indicates a promising candidate for further evaluation as a nematocide. Future work will focus on a structure-activity relationship investigation of this chemical scaffold, a toxicity assessment of potent analogues and a mechanism/mode of action investigation.

## 1. Introduction

Parasitic roundworms (nematodes) cause infections and diseases (nematodiases) in humans and animals that have a major adverse socioeconomic impact worldwide [1,2]. In humans, nematodiases disproportionately affect poverty-stricken communities, with hookworm disease, ascariasis, trichuriasis and strongyloidiasis, for example, being classified by the World Health Organization (WHO) as some of the most neglected tropical diseases [3]. From a global, agricultural perspective, productivity losses in livestock animals caused by nematodes are estimated at tens of billions of dollars [4]. Most losses in these animals relate to nematodes (order Strongylida) of the gastrointestinal and respiratory tracts. One key representative is *Haemonchus contortus* (family Trichostrongylidae), also known as the ‘barber’s pole worm’, which causes haemonchosis—a disease of ruminant livestock, such as sheep and goats. Haemonchosis is characterised by anaemia, bottle jaw, lethargy, poor wool/milk production and/or sudden death [5,6]. *H*. *contortus* is transmitted orally via a direct life cycle [7]; animals become infected by ingesting infective third-stage larvae (L3s), which exsheath in the forestomachs and develop, via fourth-stage larvae (L4s), to dioecious blood-feeding adult worms in the abomasum, where they reproduce, with females releasing eggs via faeces into the environment [7].

Treatment with anthelmintic compounds is an important component of the control of trichostrongyloid nematodes [8]. Compound classes include benzimidazoles, imidazothiazoles, macrocyclic lactones, salicylanilides, amino-acetonitrile derivatives and spiroindoles, which are commercially available to combat parasitic nematodes, including *H. contortus* [6,9]. However, the excessive and frequent use of these compounds has led to widespread resistance (including multidrug resistance) to all available classes, with the exception of the spiroindole derquantel [10,11]. Resistance develops rapidly, usually emerging within 5–10 years following the introduction of a compound on to the commercial market [12,13]. Whilst non-chemotherapeutic methods of parasite control, such as grazing management, biological control or vaccination, could prevent anthelmintic resistance, no such method appears to be highly effective without the complementary use of anthelmintic compounds [6,14].

Although the rate of discovery and development of novel anthelmintics has been relatively slow, with only monepantel [15] and derquantel [16,17] entering the commercial market since 2009, there is an impetus to discover novel chemotherapeutics with mechanisms of action against parasitic nematodes that differ from those currently commercially available. Recent progress using moderate- to high-throughput, whole-parasite screening assays has enabled the discovery of a range of synthetic and natural compounds with anthelmintic activity (reviewed by [18,19]). This progress has been enabled by the availability of diverse, well-curated compound libraries from philanthropic and commercial partners, including Compounds Australia (Griffith Institute for Drug Discovery, Australia), Johnson & Johnson and Medicines for Malaria Venture (MMV; Geneva, Switzerland) [18,20,21,22,23,24,25,26,27,28,29,30,31]. Some previous work, in collaboration with MMV, identified an approved pesticide (tolfenpyrad) and two kinase inhibitors (SNS-032 and AG-1295) with anthelmintic activity against *H. contortus* within the *Pathogen* (*n* = 400 compounds) and *Stasis (n* = 400 compounds) Box’ collections. Both tolfenpyrad and AG-1295 underwent chemical optimisation, structure-activity relationship (SAR) and/or toxicity studies, although a safe lead compound was not attained [27,32,33]. To continue the pursuit of a new and effective anthelmintic entity, we extend our discovery effort, together with MMV. We sourced the newly assembled ‘*Pandemic Response Box’*, which contains 400 structurally diverse and well-curated compounds with activities against key pathogenic bacteria (*n* = 201 entities), funguses (*n* = 46) and viruses (*n* = 153), including highly significant pathogens, such as the bacterium *Staphylococcus aureus* and the virus Influenza A.

In addition to the availability of chemical libraries through collaborations with organisations such as MMV, progress in ‘multi-omics’ research has complemented anthelmintic drug discovery—with significant advances in the understanding of the *H. contortus* genome, transcriptome, proteome and lipidome [34,35,36,37,38,39,40,41,42,43]. The use of these multi-omics advances provides a promising pathway to elucidate the mechanisms of action of novel anthelmintic compounds in *H. contortus*. In addition, the closely related free-living nematode, *Caenorhabditis elegans*, provides a powerful comparator for such mechanistic studies [44,45,46]. The genome and transcriptome of *C. elegans* has been comprehensively annotated and made publicly available from WormBase (www.wormbase.org [47]; accessed on 15 September 2021). Despite being a free-living nematode, *C. elegans* belongs to the same evolutionary clade (V) as *H. contortus*, with these two species sharing 7361 one-to-one orthologous genes [34,35,39,48]. *C. elegans* is a powerful surrogate for *H. contortus* and can be used to infer the functions of orthologous gene or protein targets [45,46,48]. Hence, the identification of novel anthelmintic compounds via high-throughput screening, complemented with these tools for elucidating mode(s) of action, creates a pathway toward anthelmintic drug discovery.

Here, we (i) undertook a whole-organism, phenotypic primary screen of compounds from the *Pandemic Response Box* against *H. contortus* and *C. elegans* to identify active compounds; (ii) assessed the potency of anthelmintic activity of (hit) compounds; and (iii) evaluated the toxicity of these hit compounds against HepG2 cells, in order to identify candidate compounds for medicinal chemistry optimisation.

## 2. Results

### 2.1. Primary Screen Identifies Three Compounds with Anthelmintic Activity

**Figure 1:**
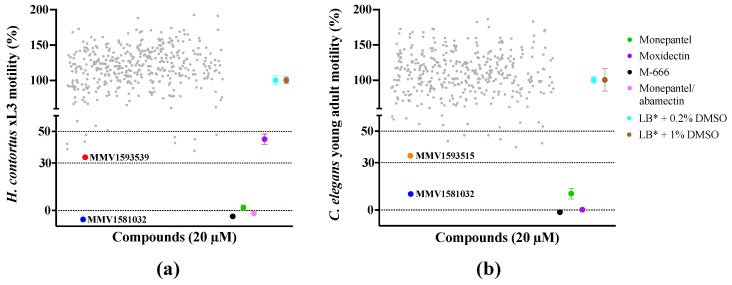
Results of the primary screen of the Medicines for Malaria Venture (MMV) *Pandemic Response Box* (*n* = 400) against (**a**) exsheathed third-stage larvae (xL3s) of *Haemonchus contortus* and (**b**) young adults of *Caenorhabditis elegans* with reference to four distinct control compounds (monepantel, moxidectin, M-666 and monepantel/abamectin) and negative (LB* + DMSO only) controls. All test and positive control compounds were tested at 20 µM. Each grey dot represents an individual test compound. Mean ± standard error of the mean (SEM) indicated for negative and positive control compounds (four data points for each positive control; 24 data points for LB* + 0.2% DMSO; eight data points for LB* + 1% DMSO).

Two compounds (Figure 1a) had marked activity and induced phenotypic changes in *H. contortus* xL3s: MMV1581032 reduced xL3 motility by ≥100% at 90 h, inhibited larval development and induced a ‘straight’ (*Str*) phenotype after seven days; MMV1593539 reduced larval motility by 66%, inhibited larval development and also induced a *Str* phenotype. Two compounds had marked activity on *C. elegans* in the transition from the L4 to the adult stage (Figure 1b): MMV1581032 and MMV1593515 inhibited larval motility by 90% and 66%, respectively, at 40 h. Thus, MMV1581032 reduced the motility of both *H. contortus* and *C. elegans* at a concentration of 20 μM. Throughout the primary screen, the Z’-factor was consistently between 0.74 and 0.86.

### 2.2. Potency and Toxicity Assessments Reveal One Promising Anthelmintic Candidate

**Figure 2:**
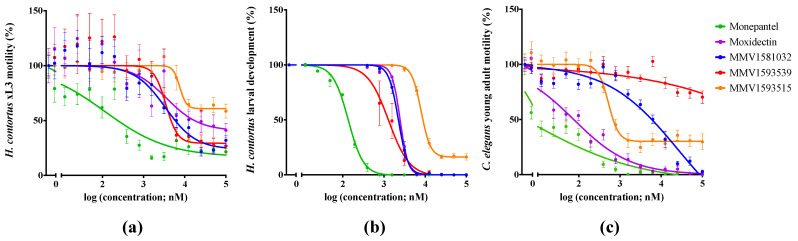
The potencies of three active test compounds (MMV1581032, MMV1593539 and MMV1593515) against exsheathed third-stage larvae (xL3s) of *Haemonchus contortus* and young adults of *Caenorhabditis elegans* with reference to two control compounds (monepantel and moxidectin). Dose-response curves show (**a**) the inhibition of *H. contortus* motility at 90 h, (**b**) the inhibition of *H. contortus* development at seven days and (**c**) the reduction of *C. elegans* motility at 40 h. Data points represent three independent experiments conducted in triplicate; the mean ± standard error of the mean (SEM).

**Figure 3:**
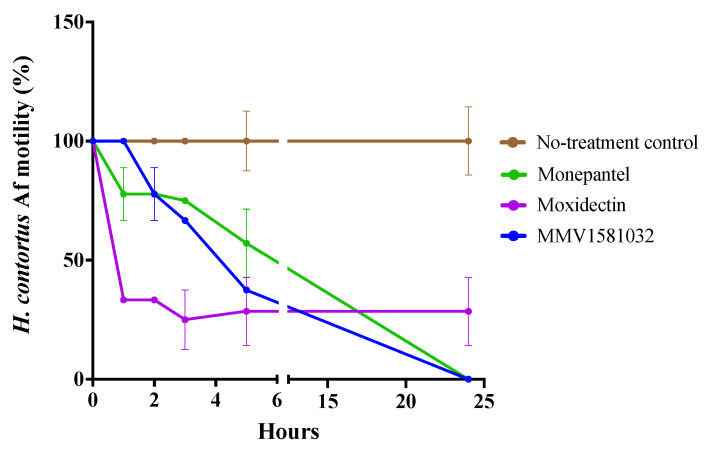
In vitro motility inhibition (%) of MMV1581032 on adult females of *Haemonchus contortus* with reference to two control compounds (monepantel and moxidectin) over a period of 24 h. Motility scores (assessed at 1-, 2-, 3-, 5- and 24-h time points; cf. [29]) for each compound were calculated, normalised with reference to the negative control (100% motility) and were recorded as a percentage. Data points represent one experiment conducted in triplicate; mean ± standard deviation (SD).

An assessment of the potency of two hit compounds to inhibit the motility of *H. contortus* xL3s was estimated following incubation for 90 h (Figure 2a; Table 1). Compound MMV1581032 displayed a half-maximal inhibitory concentration (IC_50_) of 3.4 ± 1.1 μM (maximum motility inhibition: 76%) and compound MMV1593539 had an IC_50_ of 3.5 ± 0.98 μM (maximum motility inhibition: 71%)—being comparable to that of moxidectin (IC_50_ = 7.4 ± 4.2 μM; maximum motility inhibition: 70%) in the present dose-response assay. Subsequently, the potency of each compound to inhibit larval development of *H. contortus* was assessed following incubation for seven days (Figure 2b; Table 1). MMV1581032 displayed an IC_50_ of 2.2 ± 0.68 μM, and MMV1593539 achieved an IC_50_ of 1.3 ± 0.11 μM, values that were comparable to that of moxidectin (IC_50_ = 2.4 μM). MMV1581032 was also evaluated for motility inhibition on adult females of *H. contortus* (Figure 3). MMV1581032 reduced the motility of these worms by ~0%, 22%, 33%, 63% and 100% over the time course of 1 h, 2 h, 3 h, 5 h and 24 h, respectively. Respective values for monepantel and moxidectin (Figure 3) were: 22% and 67% at 1 h; 22% and 67% at 2 h; 33% and 75% at 3 h; 43% and 72% at 5 h; and 100% and 72% at 24 h.

An assessment of the potency of two hit compounds to inhibit the motility of *C. elegans* was estimated following incubation for 40 h (Figure 2c; Table 1): MMV1581032 exhibited an IC_50_ of 7.1 ± 4.6 μM (maximum motility inhibition: 96%). MMV1593515 achieved an IC_50_ value of 0.5 ± 0.11 μM (maximum motility inhibition: 75%). Respective IC_50_ values for monepantel and moxidectin were 0.01 ± 2.0 × 10^−3^ μM and 0.04 ± 4.0 × 10^−4^ μM. Thus, MMV1581032 exhibited marked potency against both *H. contortus* and *C. elegans*.

**Figure 4:**
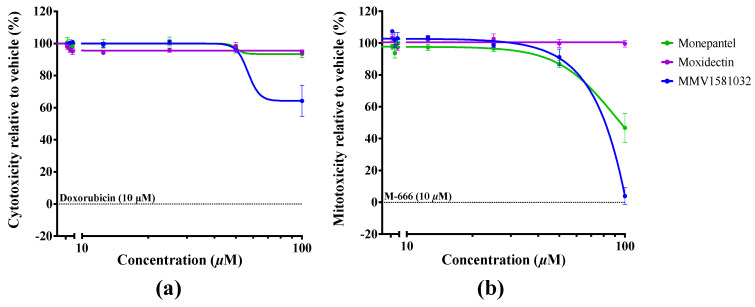
Toxicity assessment of MMV1581032, moxidectin and monepantel on HepG2 human hepatoma cells with reference to two positive controls; doxorubicin (cytotoxic) and M-666 (mitotoxic). A cell-viability assay was employed to estimate (**a**) the half-maximal cytotoxic concentration (CC_50_) and (**b**) the half-maximal mitotoxic concentration (MC_50_) after 48 h of incubation with compound. Crystal violet staining was utilised to measure the absorbance (595 nm) of treated cells compared to the negative control (100% cell viability) and baseline-corrected using respective positive controls. Data points represent one experiment conducted in triplicate; mean ± standard error of the mean (SEM).

Given the potential of MMV1581032 as a candidate, its toxicity was assessed by measuring cell death using crystal violet staining (Figure 4). This compound was shown to be neither cytotoxic (CC_50_ ≥ 100 μM) nor mitotoxic (MC_50_ = 81.1 μM) against HepG2 human hepatoma cells.

## 3. Discussion

**Figure 5:**
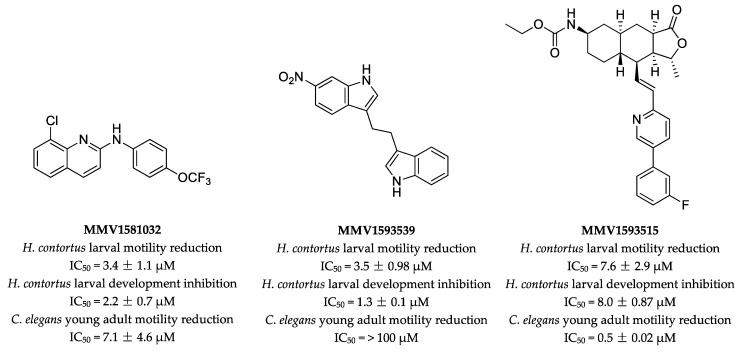
Chemical structures of MMV1581032, MMV1593539 and MMV1593515. Half of the maximum inhibitory concentration (IC_50_) values of each compound were established for the inhibition of *H. contortus* larval motility at 90 h, the inhibition of larval development of *H. contortus* at seven days and the reduction of motility of young adults of *C. elegans* at 40 h. The IC_50_ values are presented as mean ± standard deviation. Three independent assays were conducted in triplicate.

The screening of 400 diverse, drug-like molecules identified three compounds (Figure 5), designated as MMV1593539, MMV1593515 and MMV1581032, with anthelmintic activity against *H. contortus* and/or *C. elegans*. MMV1581032 was shown to be the most potent of these compounds against both *H. contortus* and *C. elegans*, with IC_50_ values of 3.4 μM and 7.1 μM, respectively, and killed adult females of *H. contortus* at 100 µM in vitro after 24 h. Thus, MMV1581032 presented as a suitable candidate for further evaluation.

Compound MMV1593539—a bis-indole derivative—was shown to be potent against *H. contortus* (3.5 μM) but not *C. elegans* (>100 μM). The reduction in both larval motility and development of *H. contortus* was comparable with that of moxidectin, but less than that of monepantel. MMV1593539 induced a ‘straight’ (*Str*) phenotype in *H. contortus* xL3s at 100 μM—a phenotype induced by other compounds, such as tolfenpyrad—a pyrazole-5-carboxamide [23]—and goniothalamin—an α-pyrone [49]. Zoraghi et al. [50] first reported MMV1593539 as a pyruvate kinase inhibitor that is selective against *Staphylococcus aureus*. Notably, genes encoding pyruvate kinase (codes HCON_00010565, HCON_00010570 and HCON_00010580; ref. [39]) have been identified in *H. contortus*. Although compound MM1593539 is selective for pyruvate kinase in *S. aureus*, it is not clear whether this is the case in *H. contortus*. However, as MMV1593539 did not show activity against *C. elegans*, the ability to perform mechanism of action studies in the N2 strain of this free-living nematode as a tool might be limited. However, other strains of *C. elegans* which are more sensitive to drugs, such as *bus-2* [51], might be more applicable. As the bis-indole structure of MMV1593539 is metabolically labile [52] and nitro functional groups (bis-amine reduction) are often associated with toxicity issues [53], the compound is not favourable from a medicinal chemistry perspective. Therefore, despite its potent anthelmintic activity against *H. contortus* larvae, SAR studies may not yield a promising lead candidate.

Compound MMV1593515—a natural himbacine derivative, also known as vorapaxar [54]—displayed a low potency (maximum motility inhibition of 38% at 7.6 µM) against *H. contortus* xL3s but high potency (0.5 µM) against *C. elegans*. Vorapaxar, an anti-platelet drug (PAR-1 antagonist), was first described in 2008 and is currently commercially available as Zontivity^®^ for the treatment of people with a history of heart attacks [54,55]. Interestingly, himbacine derivatives have been identified previously as human muscarinic acetylcholine receptor antagonists [56,57]. In *C. elegans*, the human muscarinic receptor ortholog, GAR-3, has been implicated in the regulation of muscle contraction [58]—thus, it is proposed that GAR-3 antagonism by MMV1593515 is responsible for motility reduction in *C. elegans*, although a mode of action study would be needed to test this hypothesis. Despite the low potency against *H. contortus*, voraxapar might exert anthelmintic activity against other socioeconomically important parasitic nematodes of the order Strongylida (e.g., species of *Ostertagia*, *Teladorsagia, Trichostrongylus*, *Cooperia* and/or *Ancylostoma*), given the promising potency against *C. elegans*, which is within the same evolutionary clade (V) as the Strongylida [59,60]. However, an SAR investigation utilising MMV1593515 should consider the challenges associated with producing himbacine-derivatives. As synthesising the himbacine core requires a complex multi-step (>10 steps) process [54,56,61,62,63], synthesising MMV1593515 analogues would likely be costly and time-consuming, important factors to consider prior to embarking on medicinal chemistry optimisation.

Compound MMV1581032—a quinoline derivative, also known as “ABX464” (cf. [64,65])—affected motility (*H. contortus* and *C. elegans*) and development (*H. contortus*), leading to a *Str* phenotype at 100 μM (like compound MMV1593539). The potent in vitro effect on the most pathogenic and reproductively active stage of *H. contortus* [66] encourages the optimisation of ABX464 via SAR studies and toxicity evaluation of analogues with increased potency. The quinoline scaffold of ABX464 (Figure 5) provides a sound pathway to medicinal chemistry optimisation with a wide range of diverse chemical changes possible [67]. A preliminary drug-likeness evaluation, using the Lipinski’s rule of five [68] as a guide, indicates that the ABX464 molecule fits three of four criteria; it contains one hydrogen bond donor, three hydrogen bond acceptors and has a molecular weight of 338.72 g/mol. Although the calculated logP is >5 (cLogP = 6.34; ChemDraw Ultra 12.0, PerkinElmer Informatics, Waltham, MA, USA), medicinal chemistry optimisation (by introducing hydrophilic functional groups) could address this issue. Furthermore, ABX464 analogues could be synthesised via a one-step nucleophilic aromatic substitution reaction, utilising quinoline- and/or aniline-derived building blocks. Such reagents are readily available at a relatively low cost, indicating that diverse ABX464 analogues could be produced cost-effectively and efficiently. Taken together, this information suggests that ABX464 is a promising candidate for optimisation as a nematocide.

ABX464 (i.e., MMV1581032) was first reported as a novel anti-HIV molecule [64] and is now undergoing phase 2 clinical trials as an anti-inflammatory compound for the treatment of ulcerative colitis, Crohn’s disease and rheumatoid arthritis of humans (cf. ClinicalTrials.gov, NIH; accessed on 15 September 2021). The anti-inflammatory properties of ABX464 are a consequence of RNA splicing-interference [65]. Campos et al. [64] showed that ABX464 binds to the human cap-binding complex (CBC), a protein structure located at the 5′-end of pre-mRNA transcript, which is required for efficient cellular and viral pre-mRNA splicing [64,65,69]. Upon ligand binding, CBC may undergo a conformational change, which markedly affects or alters pre-mRNA splicing [65]. As an anti-inflammatory, the binding of ABX464 to CBC has been shown to upregulate the non-coding micro-RNA, miR-124 [65,70]; miR-124 downregulates the secretion of several pro-inflammatory cytokines (IL-1β, IL-6, TNF-α); thus, the ABX464-mediated upregulation of miR-124 likely inhibits inflammation [65,70]. Although the CBC appears to bind to all 7-methylguanylate-capped RNAs (such as pre-mRNA, mature mRNA or stable long non-coding RNAs; ref. [69]), ABX464 effects a specific upregulation of miR-124 transcription [65].

Currently, the mode/mechanism of action of ABX464 in nematodes is unknown, but could be explored using genomic, transcriptomic and/or proteomic methods. Genomic and transcriptomic approaches, similar to those employed for the discovery of the target of the anthelmintic monepantel [15,45,46,71,72,73], could be applicable to parasitic nematodes more broadly. For instance, utilising a forward genetic screen in *C. elegans*, Kaminsky et al. [71] established that the gene *acr-23* encoded a target of monepantel and then identified and validated its homologue in *H. contortus* (designated *mptl-1*) [72]. The potency of ABX464 in *C. elegans* suggests that such a functional genomic approach might be useful to study this compound’s target and mode of action [45,46]. It is also expected that the use of advanced proteomic tools, such as an affinity-based pulldown and/or cellular thermal shift assays plus mass spectrometry [74], should elucidate compound-target interaction(s), underpinned by the knowledge of the somatic, excretory/secretory and phospho-proteomes for *H. contortus* [37,38,43]. Importantly, a combined, multi-omics approach is likely to be most effective towards the elucidation of mode(s) of action (cf. [40,45,46]). Although ABX464 presents as a promising candidate for mode of action studies, future work will prioritise the optimisation of this compound’s nematocidal potency and the minimisation of toxicity on mammalian cells via an detailed SAR investigation.

## 4. Materials and Methods

### 4.1. Preparation of Compounds for Screening

The *Pandemic Response Box* compound library from MMV, Geneva, Switzerland, contains 400 structurally diverse, drug-like compounds (https://www.mmv.org/mmv-open/pandemic-response-box/pandemic-response-box-supporting-information; accessed on 15 September 2021). Individual compounds were supplied at concentrations of 10 mM or 2 mM in 10 μL of (100%) dimethyl sulfoxide (DMSO). Prior to screening, compounds were individually diluted to 40 μM in sterile Luria-Bertani broth (LB; cf. [31,75]). LB was autoclaved and supplemented with final concentrations of 100 IU/mL of penicillin, 100 µg/mL of streptomycin and 0.25 µg/mL of amphotericin B (Fungizone^®^, cat. no. 15240-062, Gibco, Thermo Fisher Scientific, Waltham, MA, USA); this supplemented LB was designated LB*.

### 4.2. Production, Storage and Preparation of H. contortus

*Haemonchus contortus* (Haecon-5 strain; cf. [35]) was maintained in experimental sheep as described previously [35,76] and in accordance with the institutional animal ethics guidelines (permit no. 1714374; The University of Melbourne, Parkville, Victoria, Australia). Helminth-free Merino sheep (six months of age; male) were orally inoculated with 7000 third-stage larvae (L3s) of *H. contortus*. Four weeks after inoculation, faecal samples were collected from sheep with patent *H. contortus* infection. These samples were incubated at 27 °C and >90% relative humidity for one week to yield L3s [76], which were then collected in tap water and allowed to migrate through two layers of nylon mesh (pore size: 20 μm; Rowe Scientific, Doveton, Vic, Australia) to remove debris. Clean L3s were stored in the dark at 11 °C for up to six months [76]. Immediately prior to screening, *H. contortus* L3s were artificially exsheathed via exposure to 0.15% (*v*/*v*) sodium hypochlorite (NaOCl) for 20 min at 38 °C [76], achieving an exsheathment rate of 90%. The larvae were then immediately washed five times with 50 mL of sterile physiological saline solution by centrifugation at 500× *g* (5 min) and resuspended in LB* at a concentration of 200 exsheathed L3s (xL3s) per 50 µL (for primary screen; see Section 4.4) or 300 xL3s per 50 µL (for dose-response assays; see Section 4.6).

Adult *H. contortus* were collected from the abomasa of sheep infected for 10 weeks, washed three times in phosphate-buffered saline (PBS; pH 7.4, 38 °C) and then three times in RPMI 1640 media supplemented with 4 mM l-glutamine, 100 U/mL of penicillin, 100 µg/mL of streptomycin and 0.25 µg/mL of amphotericin B (RPMI*, 38 °C; cat. no. 11875093, Thermo Fisher Scientific, Scoresby, Victoria, Australia). Using a dissecting microscope, female and male worms were separated in RPMI* (38 °C), and female worms immediately used (within ~1 h at 38 °C) to test compounds (see Section 4.8). Females were used because they produce large numbers (~4000) of eggs per day (cf. [77,78]), giving rise to the next generation of worms.

### 4.3. Production, Storage and Preparation of C. elegans

*Caenorhabditis elegans* (N2–wildtype Bristol strain) was maintained in the laboratory under standard conditions at 20 °C on nematode growth media (NGM) agar plates, with *Escherichia coli* OP50 as a food source [79]. Gravid adult worms were collected from NGM plates, washed with sterile M9 buffer and then treated with this buffer containing 0.4% (*v*/*v*) sodium hypochlorite and 170 mM sodium hydroxide for 4–8 min at 22–24 °C to release eggs [79,80]. The eggs were then washed five times with 15 mL of sterile M9 buffer (centrifugation at 500× *g*, 2 min). After washing, the egg pellet was suspended in 8 mL of M9 buffer in a 15 mL tube and gently agitated for 24 h at 22–24 °C (room temperature) to produce first-stage larvae (L1s); 45 h prior to screening, synchronised *C. elegans* L1s were inoculated on to NGM plates containing 500 µL of *E. coli* OP50 (~3000 larvae per plate) and allowed to develop to fourth-stage larvae (L4s) at 20 °C. L4s were collected from plates and washed twice with sterile M9 buffer by centrifugation (500× *g*, 2 min) to remove *E. coli* OP50, and then resuspended in LB* at a concentration of 125 larvae per 50 µL (for primary screen; see Section 4.5) or 100 larvae per 50 µL (for dose-response assays; see Section 4.7).

### 4.4. Screening for Anthelmintic Activity against H. contortus

An established high-throughput phenotypic screening assay [31] was used to test the anthelmintic activity of compounds on *H. contortus* xL3s. Compounds were assessed for their effect on the motility of xL3s at a concentration of 20 μM in LB*. Four compounds, monepantel (Zolvix™; Elanco, Australia), monepantel/abamectin (Zolvix Plus™; Elanco, Australia), moxidectin (Cydectin^®^; Virbac, France) and compound MIPS-0018666 (abbreviated herein as M-666; cf. [81]), were used as positive controls (final concentration of 20 μM in LB*). Two solutions of LB* + 0.2% (*v*/*v*) DMSO and LB* + 1% (*v*/*v*) DMSO were used as negative controls. Test compounds as well as positive and negative controls were distributed amongst two flat-bottom 384-well microplates (cat no. 3680; Corning, Corning, NY, USA). Added to each well were 80 xL3s of *H. contortus* in 20 μL of LB* to give a final volume of 40 μL. Plates were then placed in a CO_2_ incubator (10% [*v*/*v*] CO_2_, 38 °C, >90% humidity; Forma, model no. 311, Thermo Fisher Scientific, Waltham, MA, USA). At 90 h, worm activity was captured using a WMicroTracker ONE unit (Phylumtech, Sunchales, Santa Fe, Argentina). Over a period of 15 min, disturbance of an infrared beam in individual wells was recorded as a worm ‘activity count’. Activity counts were then normalised to the positive and negative controls using the program Prism (v.9.1.0 GraphPad Software, San Diego, CA, USA) to remove plate-to-plate variation. The screening plates were then returned to the incubator (10% [*v*/*v*] CO_2_, 38 °C, >90% humidity) for an additional three days to observe compound effects on larval development. At day seven, worms were fixed with 40 μL of Lugol’s solution (cat no. 62650; Sigma-Aldrich, St. Louis, MO, USA). Larval development was identified microscopically and recorded. Additionally, a compound that induced a non-wildtype phenotype (visible microscopically at 100-times magnification) in *H. contortus* worms was recorded. A compound that reduced xL3 motility by ≥70% and/or inhibited larval development or induced an abnormal phenotype (comparative to the negative control) was recorded as a ‘hit’ compound. The performance of the assay was monitored using the Z’-factor [82] calculated using data for the negative (DMSO) and positive (M-666) control compounds on individual plates.

### 4.5. Screening for Anthelmintic Activity against C. elegans

An established assay [83] was employed to test the anthelmintic activity of compounds on *C. elegans*. Compounds were assessed for their effect on the motility of worms at a concentration of 20 μM in LB*. Three compounds, monepantel, moxidectin and M-666 were used as positive controls (final concentration of 20 μM in LB*). Two solutions of LB* + 0.2% (*v*/*v*) DMSO and LB* + 1% (*v*/*v*) DMSO were used as negative controls. Test compounds and positive and negative controls were distributed amongst two flat-bottom 384-well microplates. Added to each well were 50 *C. elegans* in 20 μL of LB* to give a final volume of 40 μL. Plates were then placed in an incubator (Heratherm, model no. IMP180, Thermo Fisher Scientific, Waltham, MA, USA) at 20 °C for 40 h. At 40 h, the motility of *C. elegans* (in the transition from L4 to the adult stage) was captured using a WMicroTracker ONE unit. Over a period of 15 min, disturbance of an infrared beam in individual wells was recorded as a worm ‘activity count’. Activity counts were then normalised to the positive and negative controls using the program Prism v.9.1.0 to remove plate-to-plate variation. The screening plates were then returned to the incubator at 20 °C. At day 5, worms were fixed with 40 μL of Lugol’s solution. Any compound that induced a non-wildtype phenotype (viewed under microscope) in *C. elegans* worms was identified. A compound that reduced worm motility by ≥70% and/or induced an abnormal phenotype (comparative to the negative control) was recorded as a ‘hit’ compound. The Z’-factor was calculated in the same manner as in Section 4.4.

### 4.6. Dose-Response Assay Using H. contortus

An established dose-response assay [76] was employed to evaluate the potency of hit compounds against *H. contortus*. Test compounds were assessed individually for an effect on the motility of xL3s (18-point, 2-fold serial dilution in LB*, 100 μM to 7.6 × 10^−4^ μM). Two compounds, namely monepantel and moxidectin (prepared in the same manner as the test compounds), were used as positive controls. A solution of LB* + 0.25% (*v*/*v*) DMSO was used as a negative control. The test compounds and positive control compounds were arrayed in triplicate across individual flat-bottom 96-well microplates (cat. no. 3596; Corning, Corning, NY, USA), with six wells on each plate containing the negative control. Added to each well were 300 xL3s of *H. contortus* in 50 μL of LB* to give a final volume of 100 μL. Plates were then placed in a CO_2_ incubator (10% [*v*/*v*] CO_2_, 38 °C, >90% humidity). The motility of xL3s was measured (as a motility index, Mi) at 90 h, and larval development established at 168 h of incubation with compound, as described previously [76]. For individual wells, raw motility indices were then normalised with reference to the negative controls. The compound concentrations were log_10_-transformed and fitted using a variable slope four-parameter equation, constraining the highest value to 100% using ordinary least squares fit model. The development inhibition and phenotypes of larvae were examined using a microscope [76], and results were analysed employing Prism v.9.1.0.

### 4.7. Dose-Response Assay Using C. elegans

A newly-established dose-response assay [83] was employed to further evaluate the potency of hit compounds against *C. elegans*. Test compounds were assessed individually for an effect on the motility of *C. elegans* (18-point, 2-fold serial dilution in LB*; from 100 μM to 7.6 × 10^−4^ μM) in the transition from the L4 to young adult stage. Two compounds, monepantel and moxidectin, were used as positive controls and prepared in the same manner as the test compounds. A solution of LB* + 0.25% (*v*/*v*) DMSO was used as a negative control. The test compounds and positive control compounds were arrayed in triplicate across individual flat-bottom 96-well microplates, with six wells on each plate containing the negative control. Added to each well were 100 *C. elegans* in 50 μL of LB* to give a final volume of 100 μL. Plates were then placed in an incubator at 20 °C for 40 h. At 40 h, worm activity was captured using a WMicroTracker ONE unit. Over a period of 15 min, disturbance of an infrared beam in individual wells was recorded as a worm activity count. Raw ‘activity counts’ for each well were normalised to the negative controls. The compound concentrations were log_10_-transformed and fitted using a variable slope four-parameter equation, constraining the highest value to 100% using the ordinary least squares fit model employing Prism v.9.1.0.

### 4.8. Motility Assay against Adult Female H. contortus

A motility assay was employed to assess the in vitro activity of a compound (MMV1581032) on adult females of *H. contortus* using an established assay [29]. The compound was added in triplicate to the wells of a 24-well plate (cat. no. 3524; Corning, Corning, NY, USA) at a concentration of 100 μM in 500 μL of RPMI*. Two positive control compounds, monepantel and moxidectin, and a negative control containing 1% (*v*/*v*) DMSO only were included in triplicates on the same plate. Four adult females were added to each of the triplicate wells containing either the test compound or the controls and placed in a CO_2_ incubator (10% [*v*/*v*] CO_2_, 40 °C, >90% relative humidity) for 1 day. A video recording (30 sec) of each well was taken at 1 h, 2 h, 3 h, 5 h and 24 h during the total incubation period to assess the reduction in worm motility, which was scored as 3 (“good”), 2 (“low”), 1 (“very low”) or 0 (“no movement”; cf. [29]). For each test or control compound, the motility scores for each of the triplicate wells were calculated, normalised with reference to the negative control (100% motility) and recorded as a percentage.

### 4.9. Evaluation of Cellular and Mitochondrial Toxicities Using HepG2 Cells

A cell viability assay was employed to evaluate the cytotoxicity and mitotoxicity of one hit compound, MMV1581032, against HepG2 human hepatoma cells (Cat. no. 85011430; Sigma-Aldrich, St. Louis, MO, USA). The test compound was serially-diluted (7-point, 2-fold serial dilution, 100 µM to 1.56 µM) in Dulbecco’s modified eagle medium (DMEM; with GlutaMax^TM^ or 4 mM L-glutamine; cat. no. 10566016 or 11966025, respectively; Thermo Fisher Scientific, Waltham, MA, USA) supplemented with 25 mM d-glucose (cytotoxicity) or d-galactose (mitotoxicity), 10% inactivated foetal bovine serum (FBS), 100 IU/mL of penicillin, 100 µg/mL of streptomycin and 0.25 µg/mL of amphotericin B (denoted DMEM*). Monepantel and moxidectin (prepared in the same manner as the test compound) were included as positive control compounds. Two compounds, doxorubicin (cytotoxic; Sigma-Aldrich, St. Louis, MO, USA) and M-666 (mitotoxic; [81]), were used as positive controls at a single concentration of 10 µM. A solution of DMEM* + 0.25% (*v*/*v*) DMSO was used as a negative control. HepG2 cells were seeded into wells of a 96-well plate in 80 µL of DMEM* (at 5.5 × 10^4^ cells per well) and allowed to adhere for 16 h at 37 °C and 5% (*v*/*v*) CO_2_ at >90% humidity prior to incubation with individual compounds, at a final volume of 100 µL. For the assessment of mitochondrial toxicity, cells were starved of serum (DMEM* without FBS) for 4 h prior to the incubation with individual compounds [84,85]. Following 48 h of incubation, cell viability was determined by crystal violet staining [86]. The absorbance (595 nm) of treated cells was normalised using the negative controls (viability: 100%) to calculate the cell viability. All compounds and controls were tested in triplicate. To determine the half-maximal cytotoxic concentration (CC_50_) and half-maximal mitotoxic concentration (MC_50_) values, compound concentrations were log_10_-transformed, baseline-corrected using a respective positive control (doxorubicin or M-666) and fitted using a variable slope four-parameter equation employing the ordinary least squares fit model using Prism v.9.1.0.

## Figures and Tables

**Table 1:** Summary of the potency assessment of hit compounds and positive control compounds (monepantel and moxidectin) on *Haemonchus contortus* and *Caenorhabditis elegans*.

Compound.	*H. contortus*			*C. elegans*
	Larval Motility (90 h) IC_50_ ± SD (μM)	Larval Development (168 h) IC_50_ ± SD (μM)	Abnormal Phenotype (168 h)	Young Adult Motility (40 h) IC_50_ ± SD (μM)
MMV1581032	3.4 ± 1.1 (76)	2.2 ± 0.7	*Str*	7.1 ± 4.6 (96)
MMV1593539	3.5 ± 0.98 (71)	1.3 ± 0.1	*Str*	>100
MMV1593515	7.6 ± 2.9 (38)	8.0 ± 0.87	*–*	0.5 ± 0.02 (75)
Monepantel	0.11 ± 0.003 (84)	0.013 ± 0.002	*Coi*	0.01 ± 0.002 (100)
Moxidectin	7.4 ± 4.2 (70)	2.4 ± 0.01	*Cur*	0.04 ± 0.0004 (100)

IC_50_ calculated from three independent assays conducted in triplicate. Value in parentheses represents the maximum motility inhibition (%). *Str*, straight; *–*, no apparent distinction from wild-type, but reduced motility; *Coi*, coiled; *Cur*, curved.

## Data Availability

Data is contained within the article.
